# Electrochemical Polishing of Austenitic Stainless Steels

**DOI:** 10.3390/ma13112557

**Published:** 2020-06-04

**Authors:** Edyta Łyczkowska-Widłak, Paweł Lochyński, Ginter Nawrat

**Affiliations:** 1 Institute of Environmental Engineering, Wrocław University of Environmental and Life Sciences, 50-363 Wrocław, Poland; pawel.lochynski@upwr.edu.pl; 2 Department of Inorganic Chemistry, Silesian University of Technology, Analytical Chemistry and Electrochemistry, 44-100 Gliwice, Poland; Ginter.Nawrat@polsl.pl

**Keywords:** electropolishing, mechanism, stainless steel, corrosion, surface roughness, microhardness

## Abstract

Improvement of the corrosion resistance capability, surface roughness, shining of stainless-steel surface elements after electrochemical polishing (EP) is one of the most important process characteristics. In this paper, the mechanism, obtained parameters, and results were studied on electropolishing of stainless-steel samples based on the review of the literature. The effects of the EP process parameters, especially current density, temperature, time, and the baths used were presented and compared among different studies. The samples made of stainless steel presented in the articles were analysed in terms of, among other things, surface roughness, resistance to corrosion, microhardness, and chemical composition. All results showed that the EP process greatly improved the analysed properties of the stainless-steel surface elements.

## 1. Introduction

Metals and their alloys, when immersed in a suitable bath and in adequate electrolysis conditions, may undergo anodic dissolution so that their surface becomes glossy and smooth [1]. The typical reactions, anodic (1) and cathodic (2), in the electrochemical polishing process are [2]:

M^0^ − ze^−^ → M^z+^ (1)

2H^+^ + 2e^−^ → H_2_ (2)

Anodic dissolution of metals and their transmission to the solution in the form of simple or complex hydrated ions may be described with the following equations:

Me + xH_2_O − ze^−^ → Me^z+^·xH_2_O (3)

Me + xH_2_O + yA^−^ − ze^−^ → [MeA_y_]^z − y^·xH_2_O (4)

The effects of electropolishing the surface of metal elements are:

- Macropolishing, i.e., removing the peaks of a height of approximately 100 µm which smoothens the surface;
- Micropolishing, i.e., removing the peaks of a height of approximately 10 µm which makes the surface glossy;
- Passivation which means the formation of a passive, oxide layer on the surface of metal [3,4,5].

Thus, during the electrochemical polishing process, the geometry of the surface is subject to changes in the micrometric range, which results in smoothing the surface. In the range below 10 µm, this results in a highly ordered, single-direction reflection of the rays of light that gives the surface mirror-like properties. 

In the electropolishing process, the surface is smoothened and lustred without using any mechanical tools, so that the superficial layer is protected against structural changes, while the crystalline structure of the core of the processed element is revealed. Moreover, the process results in uniform passivation of the whole surface of the polished element that protects it against corrosion, together with spots that are hard to access and gives the element a decorative appearance. 

Electrochemical polishing was probably first applied in industry after World War II to process various types of carbon and alloy steels [6,7]. The best technical and economic results were obtained for stainless steels, for which mechanical polishing did not lead to the same results as electropolishing. Austenitic chromium and nickel steels (e.g., AISI 304) and chromium–nickel–molybdenum (e.g., AISI 316) are widely used in heavy industry, food processing, the aviation, the automotive industry, the space industry, jewellery making as well as in sport and biomedical engineering [8,9,10]. However, not all types of metallic materials may be polished with comparable results. Good electrochemical polishing results are obtained for metals or alloys of a homogeneous and fine-grained structure which are also free from non-metallic intrusions. 

The main advantages of electropolishing include:

- Giving an aesthetic appearance;
- Obtaining good anti-corrosion properties due to the creation of the passive oxide layer containing chromium oxide Cr_2_O_3_, nickel oxide NiO, molybdenum oxide Mo_2_O_3_, and iron oxide Fe_2_O_3_ [11,12,13];
- Facilitating washing and cleaning of elements subjected to electrochemical polishing (the removal of dirt and bacteria is easier) [14];
- Removing microstresses in the superficial layer caused by processing and restoring uniform micro-hardness of the native material [15,16];
- The possibility to polish surfaces and spots that are inaccessible to mechanical polishing [17,18].

The possibility to polish soft metals and alloys and elements of a delicate, openwork structure is no less important.

Electropolishing produces a surface free from deformations without interfering with the crystalline network structure and superficial stresses. The degree of smoothening is higher the more homogeneous the alloy structure. The literature on the subject includes studies that present the influence of the polishing method on fatigue. The results of these studies demonstrated that low cycle fatigue (LCF) of electrochemical polished samples was 87% higher than that of samples polished mechanically using abrasive materials [19].

The fact that surfaces subjected to electrochemical polishing are easy to clean makes it possible to achieve a high quality of surface cleanliness and sterility. This is an important requirement in the pharmaceutical and food processing industries, because metal elements that come into direct contact with food must not cause contamination or any changes in the taste or colour of the food product.

These advantages of electropolishing support the use of this method in finishing surgical instruments and implants as well. Studies on the electrochemical polishing process conducted by Nawrat [20] led to implementing the electropolishing and chemical passivation of implants made from AISI 316L steel (e.g., screws, plates, and compressive-distractive apparati) at the “Mikromed” facility in Dąbrowa Górnicza. Pursuant to the PN-EN ISO 14630 standard, implants made from AISI 316L steel after the electrochemical polishing process were characterised by an average roughness coefficient *Ra* < 0.16 µm and very good corrosion resistance [20,21,22].

## 2. Mechanism of the Electrochemical Polishing Process

The first patent on electrolytic polishing were published by Spitalsky in 1910 [23], who worked on polishing silver in cyanide solutions.

The mechanism of the electrochemical polishing process was then analysed by numerous scientists. The first description of this process was presented by Jacguet [24,25] in the 1930s. According to Jacguet articles [24,25], the factor that has the greatest influence on surface smoothening during electropolishing is the emergence of a highly viscous layer on the anode [26]. The layer is formed on the surface of the anode as a result of the polarisation of the processed material. The anodic diffusion layer is flat on the side facing the solution and the cathode, while on the side adjacent to the anode it takes the form of the anodic surface. The layer is characterised by high electric resistance. However, this layer is thinner on micro-peaks than on micro-valleys. As a result, the peaks of roughness are dissolved first, as higher density current passes through them [26].

The process of anodic dissolution takes place in the conditions of threshold current. Due to the low electric conductivity and high viscosity of the solution forming the anodic layer that fosters the sustainable conditions of the process, the peaks of surface roughness are dissolved as a result of the differences in current density between peaks and indentations.

According to another hypothesis, also based on the presence of a highly viscous layer, the acceptors of the metal dissolution process plays an important role. The acceptors of the anodic process may be, for example, particles of water and anions that are present in the bath. Water causes hydration of metal ions, enabling them to detach from the surface, so the diffusion of acceptors towards the surface of electrochemical polished elements is decisive for the anodic dissolution process. During electropolishing, the concentration of acceptors in the vicinity of the anode is low, and the gradient of their concentration increases at micro-peaks, so that they are the first to be subjected to anodic dissolution. On the other hand, in micro-indentations the dissolution process is slightly delayed until the metal ions close to micro-peaks are released from the metal surface [27].

The manner of transporting metal ions by the said viscous layer was also studied by Datt and Landolt [28,29,30]. These authors explained the mechanism of the electrochemical polishing process by the emergence of a layer of high density, low water content, and high concentration of anodic metal ions (i.e., the aforementioned viscous layer) on the surface of the anode. According to them [28], a film emerges on the surface of the anode, consisting of dissolution products and the bath of increased density and high viscosity. It is subject to continuous transformations, as it decomposes and rebuilds itself. Considering the decomposition of this layer and the following reconstruction, one may claim that it is a dynamic system. According to the authors, the film in question is a layer saturated with salts, through which diffusion and migration transport of ions occurs in the absence of convection. Hypothetically, one may assume that in the event of the absence of concentration and temperature gradients and in a perfectly mixed bath, only migration transport of metal ions would occur.

Electrochemical polishing (EP) is also defined as a diffusion–adsorption process that also assumes the existence of a thin layer on the metal–electrolyte border that it created from the products of anodic dissolution in the suitable bath characterised by high density and viscosity. The mechanism of electropolishing based on the presence of a viscous layer in which the migration and diffusion of anodic dissolution processes occur in the direction from the anode and process acceptor (i.e., water particles and anions that are present in the anodic layer) towards the anode was also confirmed by Hryniewicz [31]. During electropolishing of stainless steel, in the electrolyte containing phosphoric acid (V) and sulphuric acid (VI), water is bound in the hydration zone of ions. Metal ions are transported into the electrolyte in the form of hydrated ions and complexes. Water deficit, which is further reinforced by electrolytic decomposition of water and the emission of oxygen, results in the conditions that foster the emergence of insoluble chemical bonds that form a thin film in the form of the so-called viscous layer. In the opinion of the author, the formation of passive layers may further hinder the transport of ions. High resistance of the electrolyte solution results in high heat emission, which fosters convection. It is also enhanced by changes in the solution density. Bagdach and Ciszewski [3,32] also describe a mechanism where a viscous layer, which is thin yet thicker than the height of micro-roughnesses, emerges on the anodic surface. This layer is characterised by higher electric resistance and increased concentration of dissolution products compared to the rest of the electrolytic solution. As a result, the current density on peaks increases, so that the dissolution rate is higher on peaks and lower in indentations. Strong adhesion of the viscous layer to the surface contributes to the stability of the described conditions near the surface of the anode.

**Figure 1:**
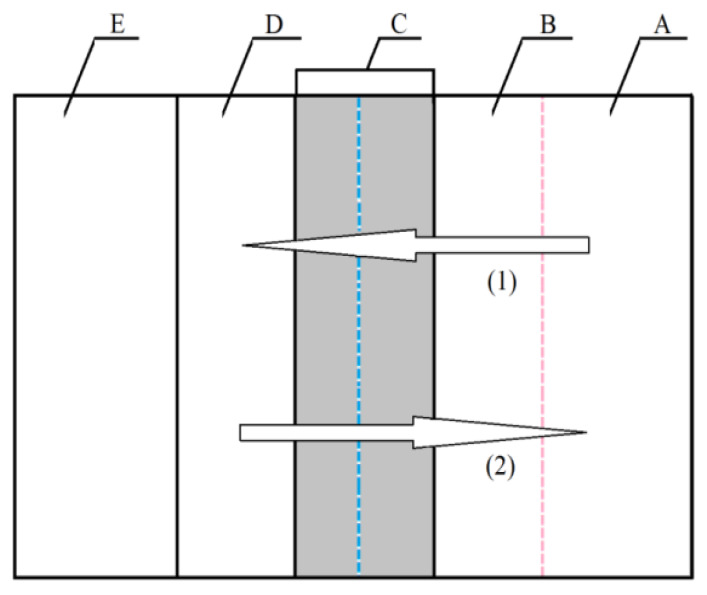
Model of anodic dissolution in the EP process (1) direction of acceptor movement, (2) direction of movement of metal ions [33,34].

A summary of the models of electrochemical polishing based on the existence of the viscous layer was presented by Lin and Hu [33,34]. The authors logically combined the hypotheses discussed above, taking into account the molecular interactions between water particles that are assigned metal ions and the presence of acids and additives that increase the viscosity of the solution. The authors divided the layer that was formed between the anodic surface (substrate E) and the depth of the solution (layer A) into three zones (B, C, and D, Figure 1).

B refers to the zone which is a thin layer formed on metal ions and acceptors that were previously adsorbed on the anode. Zone C is a layer consisting of salt solution that contains the bath and metal ions from the D layer and acceptors from layer B. The D layer consists of accumulated metal ions that emerge as a result of dissolution of metal or alloy substrate (E). It is usually a super-saturated solution. Mass transport in the electrochemical polishing process depends on the gradient of concentrations that exist in layers B and C. Metal ions migrate and diffuse from the D layer towards the depth of the solution (layer A) by electrostatic forces and thanks to the gradient of concentration. The dissolution of substrate (E) and the transport of the mass of dissolved metal ions are dominated by the micro-dimensions of the B, C, and D layers that depend on the solution used for electrochemical polishing (Figure 1). The diffusion of metal ions (E) to the bath (A) depends on the microstructure of the C and D zones. In this case, the D layer is a layer of metal salts that emerge as a result of the deposition of phosphates (V) and sulphates (VI) that are products of the reaction with bath components—phosphoric acid (V) and sulphuric acid (VI). Layer C is a liquid layer that contains concentrated metal salts that are highly concentrated, up to saturation. If the electropolishing process takes place according to the acceptor mechanism, then the diffusion of acceptors towards the anode and the migration of complexed metal ions from the anode should occur simultaneously in layer B. Hence, the microstructure of layer B is formed by the mass transport connected with these processes. The D layer disappears, while layers B and C are liquid, with a relatively concentrated layer of metal salts deposited on substrate E. This mechanism applies to chromium steel electrochemical polished in acids with a low content of water as the acceptor. When baths containing glycerine are used, polymer structures or aggregates may emerge in layer B. This results from the presence of three functional groups –OH^−^ in the particle of glycerine. These groups determine the existence of strong hydrogen bonds between them. As a result, the effect of hindering the diffusion of acceptors towards the substrate (E) and the migration of ions towards the depth of the solution (A) are reinforced. Pursuant to this mechanism, covering the substrate surface (E) by weak or strong polymer aggregate structures (e.g., particles of glycerine) influences the speed of steel dissolution.

The current convection used in the diffusion layer into the depth used is possible. However, this is a dynamic system in which the near electrode layer is constantly reproducing on the anode side and convection on the solution side. In the diffusion layer there is still a layer of adsorbed polar particles that adsorb onto the oxide layer, e.g., amines or multi-alcohols. The oxide film forms on the anode and due to the large shortage of water molecules in the layer diffusion, available in the solvation process of metal ions formed on the anode, which are cations and they change in the electric field towards the cathode, that is, into the depth of action. In these conditions, oxides or hydrated metal oxides accumulate, which are moved by moving the anodes far away from the thermodynamic state balance of the dissolution of metals:

Me − ze^−^ + nH_2_O → Me^z+^ ∙ nH_2_O (5)

which is characteristic of the metal etching process. It is also worth mentioning the role of adsorbing substances on the anode surface, such as amines and multi-alcohols, which shift potential after adsorption on the anodes far from the state of thermodynamic equilibrium of the reaction:

Me − ze^−^ → Me^z+^ (6)

The basic condition in the process of electrochemical polishing is a higher displacement of anode potential, far from thermodynamic conditions. Then the digestion process is not decided by active centres on the anode, such as the location of the metal atom in the anode metal crystals, and begins to play a role in the topography of the surface.

In the electrochemical polishing process, the anode is usually covered with an oxide film. There is only one case where no oxide layer is formed on the anode surface in the electropolishing process, i.e., the case of silver electropolishing in cyanide solutions. A salt film is then formed, which is fully the same as the oxide film in the required electropolishing.

Electrochemical polishing solutions require the ability to transfer electrical charges, which occurs in the results of anion and cation analysis in the electric field. Anions of oxygen data as well as anions of organic compounds migrating to the anode surface are capable of forming metal oxides as a result of the anode reaction. Only halogen anions cannot form metal oxides. Anhydrous solutions, such as perchlorate solution made from acetic anhydride, and concentrated chloric acid (VII) also contain chlorate (VII), ClO_4_^−^ and acetate anions, CH3COO^−^ which may be involved in the formation of oxide oxygen. Acetic anhydride can react with metal anodes with metal oxide and metal acetate. For structures forming bivalent Me^2+^ ions, a hypothetical reaction can be used:

(CH_3_COO)_2_O + 3Me − 2e^−^ → MeO + Me^2+^ + (CH_3_COO)_2_Me (7)

This reaction is much more complicated, because it results in high-molecular complex substances that have the nature of surface-active substances capable of adsorbing on the anode surface.

The presence of micro-peaks and micro-indentations of the surface during the polishing process is attributed to local differences in the rate of dissolution of the structural elements of stainless steel. The higher the difference in the rate of dissolution of specific structural components, the more uneven and rough the surface will be after electropolishing [35].

If the solution contains substances that form complex compounds with ions of the dissolved metal, then the adsorption and diffusion of the emerging complex compounds may play a significant role in the mechanism of the electrochemical polishing process. As complex compounds usually have high molar mass and a complex structure, their mobility is usually low. Polishing alloy steels in phosphate(V)–sulphate(VI) baths involves the emergence of ions [Fe(PO_4_)_3_]^6−^, [Fe(HPO_4_)_3_]^3−^, and if the bath contains oxalic acid: [Fe(C_2_O_4_)_3_]^3−^ [20].

Another known hypothesis is based on the presence of an oxide layer that emerges on the surface of the electropolishing anode in the conditions of anodic passivation. This layer is labile in a strongly acidic environment and it is in a state of dynamic balance as a result of two contradictory processes: the electrochemical formation of the oxide and its chemical degradation. The oxide layer conducts current and is characterised by variable thickness that depends on the duration and the shape of the anodic surface. As water particles and anions may access spots located on peaks of the surface more easily, the rate of chemical reactions increases which, in turn, leads to decreasing thickness of the oxide layer and increased speed of the electrochemical reaction. It should be emphasised that the emergence of oxides that form the passive layer is facilitated on the borders of grains and in spots of network defects of the metal. As a result, the structure of the processed metal or alloy influences the smoothness of the electrochemical polished element [36].

The course of the electrochemical polishing process may also be analysed in terms of the energy needs of the phase of ionisation of atoms of the dissolved metal. The energy required for the ionisation of a superficial atom during anodic dissolution is the lowest on peaks and the highest in surface indentations.

**Figure 2:**
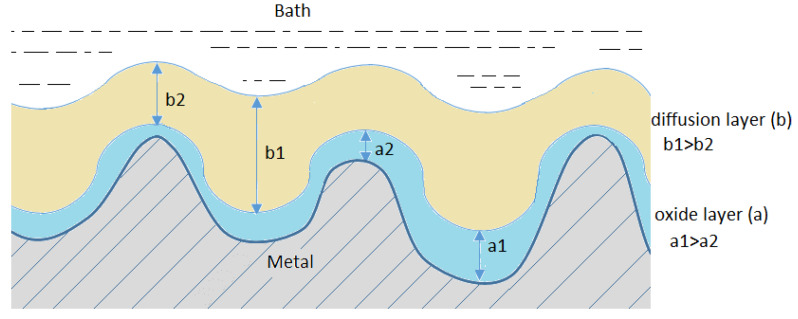
Mechanisms of the electropolishing processes.

The high number of various hypotheses concerning the mechanism (Figure 2) of smoothening and shining the surface in electropolishing results from the complexity of the process.

However, apart from the diffusion layer, also known as the viscous layer, that emerges near the anode, an oxide layer usually also emerges directly on the surface of the anode. A common phenomenon resulting from the simultaneous formation of both these layers is the process of diffusion of water particles and the migration of anions towards the surface of the anode. These substances are necessary in electrochemical formation and chemical dissolution of the oxides that constitute the passive layer. Due to the competitiveness of both these processes, even small differences in the rate of migration and diffusion of the acceptors of the dissolution process may lead to significant differences in the rate of dissolution of individual elements of the anode surface structure.

## 3. Description of the Technological Electrochemical Polishing Process

The important factors that influence the appearance of the polished surface are current density, the composition and concentration of electrolyte, mixing and bath temperature [37,38]. Current density is the ratio of the total current *I* (A) to the total surface *S* (dm^2^). Excessively high current density may cause intensive emission of oxygen, which may result in the emergence of surface defects. The type of current, including whether it is direct current or pulsating, also influences the quality of the process [3]. The composition and concentration of electrolyte, current density, and mixing rate are complementary. The conductivity of the bath depends on the concentration of its components and, as a consequence, on the density of the electrolyte. Increased temperature significantly influences the conductivity of the solution and the decrease in the clamp voltage of electrolysis. If the viscosity of the solution increases (as a result of an addition of lactic or oxalic acid or glycerine), the content of adsorbed particles on the surface of the electrochemical polished metal may increase, eventually resulting in decreased roughness.

During electropolishing, the processed element is immersed in the bath and connected to the positive pole of a direct current source (the anode). Inside the tank, cathodes are placed, which are connected to the negative terminal of the current source. The cathodes and anodes placed in electrolyte create an electric cell. In industry, tanks made from steel sheet metal layered with lead are usually used, or alternately tanks made from stainless steel and cathodes made from lead or stainless steel. The electric couplings of the hooks used for electrochemical polishing steel elements are typically made from copper wire. The electrochemical polishing process requires access to proper ventilation.

**Figure 3:**
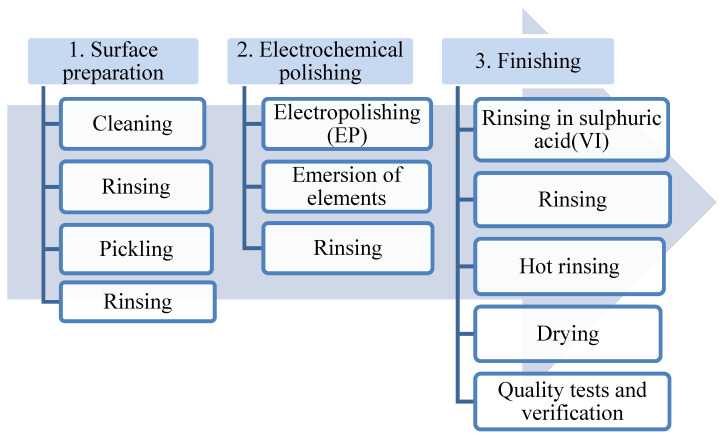
Technological diagram of a typical electrochemical polishing process.

The best results of the electropolishing process are obtained after performing three basic stages of the procedure (Figure 3):

1. Preparing the surface (removing dirt that may interfere with the electrochemical polishing process);
2. Electrochemical polishing (softening sharp edges and electrochemical polishing);
3. Final processing (rinsing and removing remains of the bath, drying the metal surface).

Figure 3 presents a technological diagram of a typical electrochemical polishing process.

Rinsing stainless-steel elements with diluted sulphuric acid (VI) additionally helps to clean the surface of the element of contaminants that may emerge from electrochemical polishing. After each stage, the processed elements are rinsed with demineralised water.

## 4. Baths and Parameters of the Electrochemical Polishing Process of Stainless Steel

The electrolyte plays several important roles:

- It is the medium in which chemical processes take place;
- It enables the transport of electric load in the solution;
- Eemoves the products of anodic dissolution from the processing zone.

The proper selection of components of the bath used for electropolishing on a laboratory and industrial scale is an important issue [39,40]. In order for the electrochemical processing to bring the desired technological and economical results, it is necessary to select such bath composition that will be adequate to the chemical composition and structure of the processed material. As attempts to determine the appropriate composition of the electrolyte are very time-consuming, baths, which are of great practical importance, are usually protected by patents.

Additionally, the scope of application of certain electrolytes is limited by their corrosive influence on the equipment and the processed element as well as harmful impact on humans and the environment [41].

In order for the thin, viscous layer to be created from the products of anodic dissolution on the anodic surface in the electrolyte it is required to maintain a low concentration of iron and chromium ions. For this purpose, after preparing the bath, it is “worked” to obtain a specific, low concentration of these ions in the solution.

During electropolishing, the concentration of metal ions in the electrolyte increases and its viscosity changes. The content of dissolved iron in the bath influences the specific weight, acidity and viscosity of the solution and the current efficiency. In practice, it is known that at 3% weight (approximately 70 g Fe/dm^3^) content of this metal in electrolyte, the electrochemical polishing process is stopped. In such event, the bath should be regenerated or replaced [31]. The durability of electrochemical polishing baths is usually determined by the value of current load that has passed through a volume unit of the bath since the moment when it was prepared. The durability of baths, e.g., for electrochemical polishing carbon steel is not high and it ranges from 100 to 180 Ah/dm^3^. Bath maintenance usually consists in removing 30–50% of the volume of the solution after processing approximately 80 Ah/dm^3^ and then supplementing it with a fresh solution [3]. Baths for polishing chromium and nickel steel are more durable. Their maintenance consists in regular supplementing of the glossing additives (e.g., triethanolamine) in half the amount specified in the formula.

The main components of solutions used for electrochemical polishing stainless steel are sulphuric acid (VI) and orthophosphoric acid (V) [42]. Sulphuric acid (VI) ensures that the electric conductivity of the bath is appropriately high, while phosphoric acid (V) is responsible for the properties of the anodic layer on the polished metal surface [43]. These acids contribute to the smoothening of the surface during electrochemical polishing and they are used to reduce the contamination of the bath (for regeneration). During electropolishing of stainless steel, the components of the steel are dissolved: iron, chromium and nickel on the anode:

Fe → Fe^3+^ + 3e^−^ (8)

Cr → Cr^3+^ + 3e^−^ (9)

Ni → Ni^2+^ + 2e^−^ (10)

2H_2_O → O_2_ + 4H^+^ + 4e^−^ (11)

Hydrogen is emitted on the cathode:

2H_3_O^+^ + 2e^−^ → 2H_2_O + H_2_ (12)

The review of literature on the surface processing of metals revealed that elements made from chromium and nickel steel may be electrochemical polished with baths that contain sulphuric acid (VI), orthophosphoric acid (V) [44], triethanolamine [45,46,47] and ethylene glycol, oxalic acid, and acetanilide [21]. Stainless steel may also be electrochemical polished in a solution consisting of sulphuric acid (VI) and citric acid, as well as of sulphuric acid (VI), phosphoric acid (V) and lactic acid [48,49]. Some other additives include: 2-amino-2-methyl-1-propanol [50], ethanolamine, diethanolamine and triethanolamine [5,51], glycerine [34], and natrium phosphate [52]. Until recently, chromic acid (VI) was also used. It was added in form of chromic acid anhydride (VI), i.e., chromium oxide (VI) [53,54,55,56]. Currently, it is used more rarely due to the fact that chromium (VI) compounds are harmful for the environment. Abbot [57], in his studies, proposed a bath consisting of ionic liquids of ethylene glycol and choline chloride instead of baths made from water solutions of acids. ChCl:EG mixtures can be a potential alternative to sulphuric/phosphoric acid-based electrolytes for electrochemical polishing stainless steel. The mechanism of electropolishing in aqueous acid solutions is different from that in the ChCl:EG mixture. The removal of the oxide is slower and more potential dependent than in aqueous solutions. When the oxide has been removed from the surface, the metal atoms are oxidised and the treatment process is controlled by mass transport limitations. When electrolytic polishing stainless steels on an industrial scale, only aqueous solutions are used, which are concentrated solutions of phosphoric and sulfuric acids. Optionally with the addition of organic substances such as glycerin, triethanolamine.

Eliaz, Nissan, and Sojitra [58,59] presented the process of electrochemical polishing implants made from 316L steel in solutions that are less harmful for the environment, i.e., in baths consisting of sulphuric acid (VI) 96% (50% vol.) and phosphoric acid (V) 85%, (50% vol.). Haïdopoulos and Zhao [60,61] conducted research on a bath consisting of glycerine 99% (47–50% vol.), phosphoric acid (V) 85% (35–42% vol.) and distilled water. These baths were used to electropolish stents made from 316L steel. Before electrochemical electropolishing, the samples were pickled in baths consisting of nitric acid and hydrofluoric acid. The process parameters for the first bath were: temperature 75 °C, current density 0.44 A/cm^2^, duration 3 min. The second bath worked at a temperature of 90–95 °C, the current density was 1.2 A/cm^2^, and time 1–10 min. Sample baths and parameters of the electrochemical polishing process of stainless steel are presented in Table 1.

The surface roughness of stents electrochemical polished in the first bath, after pickling in a solution consisting of hydrofluoric acid and nitric acid, was approx. *Ra* = 250 nm [59], while the roughness of stents that had been electrochemical polished in the second solution was *Ra* = 120.52 ± 25.65 nm. After pickling in hydrofluoric and nitric acid was *Ra* = 126.07 ± 37.13 nm [61]. Acid pickling in a bath consisting of hydrofluoric acid and nitric acid was carried out to remove slag and metal oxides attached after other types of surface processing (for example, laser micromachining) before electrochemical polishing. Electropolishing stents in the first solution resulted in a surface roughness *Ra* = 14.77 nm for the selected working parameters, while roughness after electrochemical polishing in the second bath was *Ra* = 13.13 nm. Weight loss for the first bath after pickling was 7.43%, while after the electrochemical polishing process 17.99%. In the second case, weight loss after pickling was 7.7% and 16.7% after electrochemical polishing.

## 5. Electrochemical Polishing and Other Methods of Surface Processing

Finishing is the final phase of the whole surface processing operation. Its aim is to obtain a suitably high quality of the processed element, to comply with the required technological specifications concerning the accuracy of dimensions and shape, and surface roughness. Mechanical polishing and electrochemical polishing are applied in order to obtain an appropriately smooth surface (e.g., polished surfaces of metal mirrors, ornamental elements, medical instruments, and implants).

Based on the literature, it was determined that the selection of an adequate surface processing method depends on the size and internal structure of the elements. In many cases, electrochemical polishing is preferred for the use of objects with complex shapes, because mechanical polishing is very difficult. For example [60,61], electropolishing leads to smoothing of samples of complex shapes (laser cut stents and endovascular stents) and is comparable to commercially available stents. Electropolishing does not deform the microstructure of stents; it improves it to a homogeneous structure and smooth, defect-free and contamination-free surface. Lukas Löber [62] in his studies presented a manner of surface processing of elements made from 316L steel of the dimensions of 10 × 10 × 10 mm. In order to reduce roughness, the processed elements manufactured in the SLM (Selective Laser Melting) technology were subjected to the following mechanical processing: blast cleaning, grinding, mechanical polishing as well as electrochemical polishing and electro-plasmatic polishing (Table 2).

For example, test results for processing elements manufactured from 316L steel in SLM technology, discussed in the study [62], are presented in Table 2. Positive effects were obtained after combining mechanical polishing with electropolishing: the roughness parameter *Ra* decreased from 15.03 µm to 0.21 µm and after mechanical polishing combined with electro plasmatic polishing, after which the roughness was 0.12 µm. The author of the quoted paper also mentioned the possibility to process elements after the SLM process by means of chemical pickling. Unfortunately, the level of surface roughness obtained after only mechanical initial processing (e.g., grinding) was unsatisfactory.

Table 3, based on reviewing the literature, contains the parameters of the electrochemical polishing process presented in the subject literature:

Current—*I* (A), voltage—*U* (V), current density—*j* (A/cm^2^, A/dm^2^), electric load—*q* (Ah/cm^2^), temperature—*T* (°C, K), time—*t* (s, min, h), equals the composition of the electrochemical polishing baths and main results of the tests. The specific publications mainly analysed the following: surface roughness (with use of AFM or profile meter), chemical composition of the surface (EDX, AES, and XPS), surface gloss, corrosion resistance, chemical composition of the electropolishing bath, adhesion of cells/bacteria to the processed surface, and the hardness of sample surfaces. Experiments were conducted for stainless steels: 304, 316L, 316LVM, 430, and duplex 2205. Measured 2D and 3D roughness indicators:

- *Ra* (µm, nm)—mean arithmetic deviation of surface profile from the average line measured along the measurement or elementary section;
- *Rq* (µm, nm)—root mean square deviation of surface profile from the average line measured along the measurement or elementary section;
- *Rz* (µm, nm)—maximum height of roughness from the average line measured along the measurement or elementary section;
- *Rp* (µm, nm)—maximum profile peak height;
- *Rv* (µm, nm) —depth of the deepest profile indentation;
- *Sa* (µm, nm) —mean arithmetic deviation of surface roughness from the reference plane;
- *Srms* (µm, nm) —root mean square surface roughness.

Corrosion tests indicators:

- *OCP* (V)—open circuit potential;
- *Ecor* (mV, V)—corrosion potential;
- *Epit* (V)—pitting potential;
- *Vp* (mm/y)—corrosion rate.

Raw/ground samples were designated as SS, samples after mechanical polishing as after MP, after electrochemical polishing after EP, after magnetic electrochemical polishing—after MEP.

In the studies presented above, the surface quality and the surface smoothening effect were usually controlled by analysing the roughness profile *Ra* (nm, µm). After electrochemical polishing, it was possible to obtain lower *Ra* values for raw samples and samples previously subjected to mechanical or chemical processing. Electropolishing lowered the surface roughness *Ra* of samples of implants made from 316L steel from 18.4 ± 4.5 nm (AFM) and 183.1 ± 80.6 nm (profile meter) to 2.1 ± 0.8 nm and 76.2 ± 67.9 nm [58]. Chemical pickling and mechanical processing also reduced the roughness parameter *Ra*, compared to the initial sample—SS: *Ra* = 120.52 ± 26.65 nm, after pickling: *Ra* = 126.07 ± 37.13 nm, after relaxing: *Ra* = 142.71 ± 26.20 nm, after EP*: Ra* = 13.13 ± 1.56 nm [61]. Lower *Ra* parameter of *EP* samples (*Ra* < 0.11 µm), after previous pickling (*Ra* = 0.37–0.43 µm) compared to the initial samples (*Ra* = 0.17–0.25 µm) was obtained in publication [17].

The chemical composition of samples was mainly analysed using X-ray photoelectron spectroscopy (XPS). The sample surface was analysed both in the initial state, directly after processing or after additional cleaning with Ar^+^ ions. Testing revealed that the composition of the passive layer after EP was enriched with chromium (chromium oxides and hydroxides) whose content, for the SS sample, was: at.% Cr = 5.7% at. and after EP*:* at.% Cr = 11.5% at. [60]. A similar correlation was obtained in other studies, where an increase in at.% Cr was observed from 16% (mechanically processed sample) to 20% after EP, along with a decrease in the Fe (iron oxides) content in the top layer: after mechanical processing: at.% Fe = 18%, after EP*:* at.% Fe = 10% [79]. The low ratio of iron oxides content to the total chromium oxide and hydroxide content may contribute to improved corrosion resistance of the passive layers obtained as a result of EP.

Qualitative analysis of the chemical composition of the surface was also performed using energy-dispersive X-ray spectroscopy (EDS) [76]. The chromium content in the passive layer increased from 18.83 wt.% to 19.33 wt.%, after electrochemical polishing. The tests also analysed surface gloss before and after EP. After initial preparation of samples (grinding) gloss *G* = 400, while after EP the gloss increased to *G* = 1700–2500.

Corrosion resistance was determined in potentiodynamic studies [59,71,72,74,75]. Based on results, it was determined that the corrosion resistance of 304 and 316L stainless steel improved significantly. High chromium content in both these types of steel enables the formation of the passive layer that protects the material against a corrosive environment on the surface of elements. Polarisation measurements revealed a noticeable improvement in pitting corrosion resistance and a shift in the pitting potential towards higher potential values. For samples after EP, *Epit* was 0.57 V and it was noticeably higher than for pickled samples *Epit* = 0.38 V and raw samples *Epit* = 0.30 V (versus satd. calomel electrode) [71,72]. In other studies, *Ecor* for mechanically processed surface was *Ecor* = −0.533 V, while after EP*: Ecor* = −0.324 V [74,75]. A similar correlation was found in study [59]. After pickling *Ecor* was −0.326 V, and after electrochemical polishing *Ecor* = −0.173 V. The presented research results allow us to state that electropolishing improves corrosion resistance compared to samples that were raw, pickled or mechanically processed.

Another interesting research project analysed the adhesion of blood cells to the surface of samples from 316L-SS steel, after electrochemical polishing, at direct current voltage *U* = 2.5, 4 and 10 V (*T* = 65–70 °C, *t* = 3 min) [64]. Along with the decrease in *SS* surface roughness*: Ra* = 188 ± 9 nm, EP (2.5 V) *Ra* = 107 ± 6 nm, EP (4 V) *Ra* = 77 ± 4 nm, EP (10 V) *Ra* = 97 ± 11 nm and increase in corrosion resistance: SS*: OCP* = 0.34 V, EP (2.5 V) *OCP* = 0.29 V, EP (4 V) *OCP* = 0.18 V, reduced adhesion of blood cells to the surface of 316L steel was observed for samples after EP: at 2.5 V by 71%, at 4 V by 89% and at 10 V by 93%. This study permits us to state that electropolishing significantly contributes to the reduced adhesion of blood cells to the surface of the processed elements (stents), which has a significant influence on thrombogenicity.

Electrochemical polishing also reduces the adhesion of bacteria to the surface of stainless-steel elements used in heavy industry, food industry, pharmaceutical and medical industry [91,92,93,94]. The adhesion of bacteria to the surface of devices (the formation of biofilm) may result in the contamination of the product by penetration of pathogens from the biofilm [95,96,97] and in damage or destruction of the stainless-steel surface [98,99]. After EP, the number of bacteria on the surface of stainless steel was reduced by 79% and 94% [91]. Electrochemical processing may have a significant influence on enhancing resistance to the formation of bacterial biofilm on the surface of devices [100].

The electropolishing process also influences the microhardness of the processed elements [89,90]. The microhardness of raw samples was 168.27 kg/cm^2^, after sanding −283 kg/cm^2^, while after sanding and EP < 205 kg/cm^2^. The above results indicate that the microhardness of material subjected to mechanical processing is higher than after electrochemical polishing. Sanding causes cold-work hardening, which has a significant influence on the microhardness results. Compression after mechanical processing may be partly removed by electropolishing. Samples after mechanical processing were characterised by higher microhardness than samples after mechanical processing and electrochemical polishing.

Surface defects were presented after the electropolishing process in industrial conditions [46]. A bath containing H_3_PO_4_ and H_2_SO_4_ and triethanolamine contaminated in industrial conditions was used for the tests, and the iron ions contamination was about 3% by wt. Samples with an area of approximately 33.3 dm^2^ were used in the industrial tests. For comparison, laboratory-scale tests were carried out with the area of the samples 0.4 dm^2^. The results of laboratory tests make it possible to estimate the weight loss that will be obtained in industrial conditions, taking into account the use of the same electrolyte. Due to the limitations in industrial conditions and repeated electropolishing of large-size work pieces, the current density range used was 4–8 A/dm^2^, the temperature range 35–55 °C.

**Figure 4:**
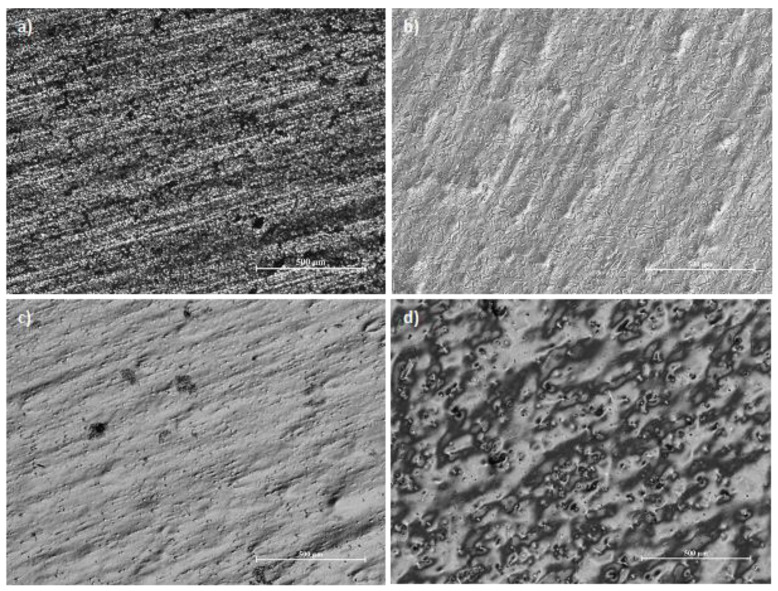
Metallographic photos of austenitic chromium–nickel stainless steel: (**a**) as-received, 2B surface finish, (**b**) high gloss surface after EP (*T* = 35 °C, 8 A/dm^2^), (**c**) edge of the sample with high-gloss surface after EP (*T* = 55 °C, *j* = 4 A/dm^2^), (**d**) internal area of the sample with orange peel effect after EP (*T* = 55 °C, *j* = 4 A/dm^2^).

The best effects were obtained for the low temperature and current density 8 A/dm^2^. The combination of low current density and high temperature resulted in an uneven polishing of the sample and “orange peel effect". On one hand the surface is polished, on the other this kind of textural irregularities are similar to the skin of an orange (Figure 4). Besides, the “orange peel effect” occurs in metals and alloys with a thick crystalline structure, because the oxide film, adsorption film and diffusion film, which shift the anode’s potential far from the thermodynamic state characteristic of the anodic etching process, are not able to overcome the effect of the privileged influence for the digestion anode places in the case of metal digestion under thermodynamic conditions, called active centres. This is the case when the surface is shiny but not smooth.

In Reference [101], defects were presented such electropolishing streaks (oxygen formation and movement). After the electropolishing process, the surface may contain certain defects, including those resulting from long-term use of electrolyte, improper current density, or local overheating of the electrolyte. Defects may be a result of non-optimal parameters and bath composition and contamination. The most common defects include orange peel effect, shadows, smudges, streaks, and uneven polishing of the material [102].

The electropolishing process parameters, bath composition and contaminations have a major role in the possibility of the occurrence of defects. Also, the selection of optimal process parameters mainly depends on the type of electrolyte used and the degree of its contamination. Undoubtedly, mixtures of H_3_PO_4_ and H_2_SO_4_ are most often used, sometimes with the addition of organic additives, e.g., glycerine. When choosing process parameters, the shape and size of electropolished samples should also be considered. Some researchers even used high temperatures 75–95 °C for electrochemical polishing of small samples. When electropolishing large-size workpieces and the need to use the lowest possible current densities, often in the range of 4–5 A/dm^2^, depending on the rate of electrolyte contamination, temperatures below 50 °C could be also used.

About the right course for the proper conduct of the electropolishing process and other electrochemical processes, decisive is the value of the electrode potential, and the process parameters must be selected so that the anode potential has the desired value. Therefore, for large-size workpieces, high values of the current supplied to the anode must be used, which requires an appropriate power supply and may cause “burns” on the workpiece being processed. The places occupied by the current contacts, of course, remain raw because there is no processing. Designing such a bath so that low current densities can be used obviously requires lower currents at the same surface size, which limits these adverse effects. Baths that require lower current densities are always better.

The paper focused on the results of electrochemical polishing of stainless-steel parameters and the effect of the process on surface roughness, chemical composition, corrosion resistance, adhesion of blood cells, adhesion of bacteria and influences the microhardness of the processed elements. The paper could help in selecting the best EP process parameter—depending on what the final part of stainless steel is being used for and which surface quality meets individual expectations.

## 6. Conclusions

The large number of hypotheses concerning the electrochemical polishing mechanism results from the complexity of the process. Apart from the anodic diffusion layer of high density and electric resistance, also called the viscous layer, thatt is formed in the proximity of the anode, an oxide layer is also formed on the surface of the anode itself. The presence of these layers limits the processes of diffusion of water particles and migration of hydrated anions and cations in the electric field. A common phenomenon that results from the simultaneous formation of both these layers is the process of diffusion of water particles and migration of anions towards the anode as well as the migration of anodic dissolution products away from the anode.

Water particles and anions, which are referred to as the acceptors of the anodic dissolution process, are necessary in the process of electrochemical formation and chemical dissolution of the oxides that constitute the passive layer. Due to the competitiveness of both these processes, even small differences in the rate of migration and diffusion of the acceptors of the dissolution process may lead to significant differences in the rate of dissolution of individual elements of the anode surface structure.

For designing the composition of the electrochemical polishing bath solution and selecting the parameters of the electrolytic polishing process, it is beneficial to assume the occurrence of three, at the same time, independent physicochemical processes:

- anodic passivation (formation of an oxide film);
- adsorption of surface-active substances;
- formation of a diffusion layer with increased viscosity and density and reduced water content concerning the rest of the solution.

The most popular additions to phosphate–sulphate solutions used as electropolishing baths are glycerol (glycerine) or chromic acid anhydride, CrO_3_. Glycerol increases the viscosity and density of the solution, which increases the thickness of the diffusion film and the greater distance of the anode potential from the thermodynamic value. Glycerol also reduces the relative concentration of water in the near-anode area and as a substance with active oxygen atoms forms an adsorption film on the surface of the anode. Chromic anhydride also increases the viscosity and density of the solution but also has oxidizing properties, which facilitate the formation of the oxide film. Of course, chromium anhydride and glycerine should not be added at the same time, as there would be a reaction between the oxidant (chromic acid) and the reducing agent (glycerine).

The main method of modifying the surface (preceding mechanical polishing and electrochemical polishing) or the final processing of metal elements is abrasive processing. Mechanical processing (machining, blast cleaning, or tumbling) cannot ensure the desired smoothness and visual properties of elements of a complex internal structure. After electropolishing, samples are characterised by lower roughness *Ra* and higher corrosion resistance. Electrochemical polishing reduces the adhesion of cells and bacteria to the processed surfaces, positively influencing the usability and durability of the devices. Errors in designing and assembling devices made from 304 and 304L stainless steel used in constructing technological equipment installed in wastewater treatment plants may result in a fast rate of corrosion. The advantage of electropolishing is that it may be applied both as a finishing process and after previous mechanical or chemical processing.

## Figures and Tables

**Table 1:** Sample baths and parameters of the electrochemical polishing process of stainless steel.

Material	Bath	Parameters			Source
		*j* (A/dm^2^)	*t* (min)	*T* (°C)	
**Steel AISI 304, 316**	Sulphuric acid (VI) (35 wt.%), orthophosphoric acid (V) (51 wt.%), triethanolamine 99%. (3 wt.%), H_2_O (11 wt.%)	20	12	55	[17]
	Sulphuric acid (VI) 96% (40% vol.), orthophosphoric acid (V) 85% (60% vol.), additives: ethylene glycol 99%—200 g/dm^3^, oxalic acid—200 g/dm^3^, acetanilide—200 g/dm^3^.	35–50	1–50	60	[21]
	Sulphuric acid (VI) 96% (50% vol.), orthophosphoric acid (V) 85%, (50% vol.)	15	1–3	40–75	[58,59]
	Orthophosphoric acid (V) 85% (35% vol.), glycerine 99% (50% vol.), distilled water (15% vol.)	75	1–10	60–95	[60,61]
	Base solution: orthophosphoric acid 85%: sulphuric acid (VI) 97% at a ratio from 2:1 to 3:2; (75% vol.), glycerine 99% (25% vol.)	50	1–10	30–90	[34]

**Table 2:** Results of roughness measurements for elements from 316L steel manufactured in the SLM (Selective Laser Melting) technology, after selected processing methods [62].

Processing Method	Abrasive Grit	*Ra* (µm)
SLM		15.03
Grinding	P 80	2.22
	P 240	1.15
	P 300	0.52
	P 500	0.43
Blast cleaning with glass	50–150 mm	8.85
Electrochemical polishing after SLM		15.03
Grinding and electrochemical polishing	P 80	9.28
	P 240	1.46
	P 500	0.64
Mechanical polishing and electrochemical polishing	-	0.21
Mechanical polishing and electro plasmatic polishing	-	0.12

**Table 3:** Sample parameters of the electrochemical polishing (EP) processes, bath compositions and test results.

EP Process Parameters	EP Bath	Type of Analyses/Material	Test Results	Source
*T* = 40, 50, 58, 67 °C, *U* = 2–4 V, *I* = 0.04 A, *j* = 0.15 A/cm^2^, *t* ≤ 10 min, *S* = 0.27 cm^2^, *q* ≤ 24.7 mAh/cm^2^.	H_2_SO_4_ 96%, H_3_PO_4_ 85% (50% *v*/*v*).	Surface roughness/316LVM.	Mean roughness: AFM: SS: *Ra* = 18.4 ± 4.5 nm, after EP: *Ra* = 2.1 ± 0.8 nm. Profile meter: SS: *Ra* = 183.1 ± 80.6 nm, after EP: *Ra* = 76.2 ± 67.9 nm.	[58]
*U* = 10–12 V, *I* = 1.2 A, *T* = 90–95 °C, *t* = 1 min, *S—*surface of one stent.	H_3_PO_4_ 85% (42 wt.%), glycerine (47 wt.%), H_2_O (11 wt.%).	Surface roughness/316L. stents: length 15 mm, diameter 1.6 mm, wall thickness 95 µm.	Mean roughness: (a) central area of the sample: SS*: Ra* = 120.52 ± 26.65 nm, pickled: *Ra* = 126.07 ± 37.13 nm, relaxed: *Ra* = 142.71 ± 26.20 nm, after EP: *Ra* = 13.13 ± 1.56 nm, (b) laser cutting area: SS*: Ra* = 491.26 ± 52.46 nm, pickled: *Ra* = 268.67 ± 27.7 nm, relaxed: *Ra* = 302.90 ± 23.33 nm, after EP: *Ra* = 15.01 ± 1.79 nm.	[61]
*U* = 9.5 V, *I* = 0.44 A, *T* = 75 °C, *t* = 3 min, S—surface of one stent.	H_2_SO_4_ 96%, H_3_PO_4_ 85% (50% *v*/*v*).	Surface roughness, corrosion resistance/316LVM, stents: length 16 mm, diameter 1.720 mm, wall thickness 110–115 µm. Corrosion tests were conducted in Phosphate Buffer Saline solution (PBS) at pH 7.4 and 37 ± 1 °C constant temperature. Open circuit potential was measured for 1000 s with respect to saturated calomel electrode (SCE).	Processing result: 78.10% roughness reduction: SS*: Ra* = 250 nm, after EP*: Ra* = 14.77 nm, SS: *Rq* = 97.56 nm, after EP*: Rq* = 4.895 nm, SS: *Rp* = 208.7 nm after EP*: Rp* = 32.49 nm, SS: *Rv* = 130 nm, after EP*: Rv* = 8.921 nm. Corrosion potential: -after pickling: *Ecor* = −326 mV, -after laser cutting: *Ecor* = −259 mV, -after EP: *Ecor* = −173 mV.	[59]
*I* = 6.8 A, *j* ≤ 40 A/dm^2^, *T* = 55 ± 1 °C, *t* = 4, 6 min, *S* = 20 cm^2^, *q* ≤ 40 mAh/cm^2^.	1—H_3_PO_4_ (51 wt.%), H_2_SO_4_ (35 wt.%), H_2_O (14 wt.%), 2—H_3_PO_4_ (51 wt.%), H_2_SO_4_ (35 wt.%), glycerine (3 wt.%), H_2_O (11 wt.%). 3—H_3_PO_4_ (51 wt.%), H_2_SO_4_ (35 wt.%), triethanolamine (3 wt.%), H_2_O (11 wt.%). Other organic additives: triethylamine, ethanoloamine, diethanolamine, butyldiglycol.	Surface roughness, corrosion resistance, gloss/samples from 304 steel, dimensions: 90 × 25 × 1.5 mm.	Bath 1: pickled: *Ra* = 0.35–0.38 µm, after EP: (*q* = 0.02 Ah/cm^2^) *Ra* = 0.12 µm, after EP: (*q* = 0.03 Ah/cm^2^) *Ra* = 0.10 µm, after EP: (*q* = 0.04 Ah/cm^2^) *Ra* = 0.12 µm. Gloss: (*t* = 6 min, *j* = 30 A/dm^2^, *q* = 0.03 Ah/cm^2^) = 890 ± 10 GU. Bath 2: pickled: *Ra* = 0.36–0.40 µm, after EP: (*q* = 0.02 Ah/cm^2^) *Ra* = 0.14 µm, after EP: (*q* = 0.03 Ah/cm^2^) *Ra* = 0.10 µm, after EP: (*q* = 0.04 Ah/cm^2^) *Ra* = 0.09 µm. Gloss: (*t* = 6 min, *j* = 30 A/dm^2^, *q* = 0.03 Ah/cm^2^) = 680 ± 10 GU. Bath 3: pickled: *Ra* = 0.36–0.38 µm, after EP: (*q* = 0.02 Ah/cm^2^) *Ra* = 0.095 µm, after EP: (*q* = 0.03 Ah/cm^2^) *Ra* = 0.09 µm, after EP: (*q* = 0.04 Ah/cm^2^) *Ra* = 0.079 µm. Gloss: (*t* = 6 min, *j* = 30 A/dm^2^, *q* = 0.03 Ah/cm^2^) = 900 ± 20 GU. Recommended processing parameters: bath 3, *j* = 30 A/dm^2^, *T* = 55 °C, *t* = 6 min.	[63]
*U* = 2.5; 4; 10 V, *T* = 65–70 °C, *t* = 3 min, *S* = 3.33 cm^2^.	H_3_PO_4_ 85% (60% *v*/*v*), H_2_SO_4_ 95–97% (20% *v*/*v*), glycerine 99.5 % (10% *v*/*v*), H_2_O (10% *v*/*v*).	Surface roughness, corrosion, chemical composition of surface, blood cells adhesion/316L, diameter 12.7 mm, wall thickness 2 mm. Corrosion tests were conducted in 0.16 M NaCl.	SS: *Ra* = 188 ± 9 nm, after EP: (*U* = 2.5 V) *Ra* = 107 ± 6 nm, after EP: (*U* = 4 V) *Ra* = 77 ± 4 nm, after EP: (*U* = 10 V) *Ra* = 97 ± 11 nm. Corrosion potential: SS*: OCP* = 0.34 V, after EP: (*U* = 2.5 V) *OCP* = 0.29 V, after EP: (*U* = 4 V) *OCP* = 0.18 V, Adhesion-decrease: after EP: (*U* = 2.5 V) by 71%, after EP: (*U* = 4 V) by 89%, after EP: (*U* = 10 V) by 93%.	[64]
*U* = 10 V, *T* = 60 °C, (EPO—oxygen evolution potential), *T* = 25 °C (EPBO—oxygen evolution plateau), *t* = 5 min.	1—H_3_PO_4_ 85%, H_2_SO_4_ 93%-7:3 vol. (EPO), 2—100 mL CH_3_OH, 300 mL H_2_SO_4_ 93% (EPBO).	Surface roughness, corrosion resistance, chemical composition of the surface, cell adhesion/316L Corrosion tests were conducted in Phosphate Buffer Saline solution (PBS) at pH 37 °C constant temperature. Calomel electrode (SCE) was used as the reference electrode and graphite rod was used as a counter electrode.	SS*: Ra* = 33.51 ± 5.54 nm, after EPO: *Ra* = 11.50 ± 1.61 nm, after EPBO: *Ra* = 6.07 ± 0.73 nm, SS*: Rq* = 48.28 ± 8.65 nm, after EPO: *Rq* = 14.79 ± 1.90 nm, after EPBO: *Rq* = 7.91 ± 0.73 nm. Corrosion current density: SS*: jcorr* = 56.1 mA/m^2^, after EPO: *jcorr* = 23.7 mA/m^2^, after EPBO: *jcorr* = 12.9 mA/m^2^, Corrosion potential: SS*: Ecor* = −410 mV, after EPO: *Ecor* = −353 mV, after EPBO: *Ecor* = −288 V, Linear corrosion rate: SS*: Vp* = 0.0475 mm/y, after EPO: *Vp* = 0.0182 mm/y, after EPBO: *Vp* = 0.0123 mm/y, Polarisation resistance: SS: *Rs* = 93.40∙Ωcm^2^, after EPO: *Rs* = 104.2 Ω∙cm^2^, after EPBO: *Rs* = 100.8 Ω∙cm^2^, Chemical composition of the surface oxide layer (XPS): SS*:* Fe2p = 13.89 wt.%, after EPO: Fe2p = 10.51 wt.%, after EPBO: Fe2p = 11.5 wt.%, SS*:* Cr2p = 1.86 wt.%, after EPO: Cr2p = 7.55%, after EPBO: Cr2p = 8.55 wt.%, after EPO: Cr2p3 = 5.65 wt.%, after EPBO: Cr2p3 = 1.23 wt.%, SS: O = 43.92 wt.%, after EPO: O = 36.51 wt.%, after EPBO: O = 38.75 wt.%.	[65]
*U* = 5 V, *I* = 0.6 A, *j* = 25 A/dm^2^, *T* = 50 °C, *t* = 20 min, *S* = 2.4 cm^2^, *q* ≤ 83 mAh/cm^2^.	H_3_PO_4_ (60% *v*/*v*), H_2_SO_4_ (40% *v*/*v*).	Surface roughness, corrosion resistance, chemical composition of the surface, cell adhesion/316L, 10 × 10 × 1 mm. Corrosion tests were conducted in ringer solution at 37 °C constant temperature. Silver chloride electrode (Ag/AgCl) and platinum counter electrode were used as reference electrode and a working electrode.	SS*:* AFM *Sa* = 161.34 ± 57.15 nm, after EP: *Sa* = 5.05 ± 0.28 nm, after EP + chemical processing: *Sa* = 0.96 ± 0.29%, SS*: Srms* = 206.58 ± 70.06 nm, after EP: *Srms* = 8.43 ± 0.40 nm, after EP + chemical processing: *Srms* = 1.71 ± 0.78%, Corrosion current density: SS*: jkor* = 0.921 µA/cm^2^, after EP: *jkor* = 0.61 µA/cm^2^, after EP + chemical processing: *jkor* = 0.0066 µA/cm^2^, Corrosion potential: SS*: Ecor* = −343 mV, after EP: *Ecor* = −292 mV, after EP + chemical processing: *Ecor* = −16 mV, XPS: SS*:* C = 80.2 at.%, after EP: C = 40.8 at.%, after EP + chemical processing: C = 37 at.%, SS*:* O = 19.8.2 at.%, after EP: O = 49 at.%, after EP + chemical processing: O = 44.2 at.%, Fe: after EP*:* Fe = 3.4 at.%, after EP + chemical processing: Fe = 3.2 at.%, Cr: after EP*:* Cr = 4.2 at.%, after EP + chemical processing: Cr = 6.0 at.%, P: after EP: P= 2.7 at.%, after EP + chemical processing: P = 9.2 at.%, Cell viability: approx. 80% (samples subjected to electrochemical and chemical processing).	[66]
*T* = 20–90 °C, *I* = 1.5 A, *j* = 0.75 A/cm^2^, *t* = 1–6 min, *S* = 2 cm^2^ (immersed surface)*q* ≤ 75 mAh/cm^2^.	glycerine 99% (50% *v*/*v*), H_3_PO_4_ 85% (35% *v*/*v*), H_2_O (15% *v*/*v*).	Surface roughness, chemical composition of the surface/316, sample dimensions: 15 × 10 × 1.5 mm.	AFM: SS: *Ra* = 2.2 ± 0.15 nm, ground: *Ra* = 2.3 ± 0.2 nm, Mechanically polished: *Ra* = 0.04 ± 0.01 nm, after EP: *Ra* = 0.07 ± 0.02 nm.Chemical composition–increase at.% Cr from 5.7% to after EP 10% (for *T* = 20 °C) and to 11.5% (for *T* > 20 °C). Recommended parameters: *t* = 3 min, *T* = 90 °C.	[60]
For baths 1 and 2 *EP:* *I* = 4 A, *j* = 20 A/dm^2^, *T* = 55 °C, *t* = 1–20 min, For bath 3 *EP:* *T* = 90 °C, *t* = 120 min, *S* = 20 cm^2^, *q* ≤ 66.7 mAh/cm^2^.	1: - H_2_SO_4_ 96% (35% *v*/*v*), H_3_PO_4_ 85% (60.5% *v*/*v*), triethanolamine 99% (4.5% *v*/*v*). 2: - H_2_SO_4_ 96% (40% *v*/*v*), H_3_PO_4_ 85% 60% *v*/*v*, glycol 99% 200 g/dm^3^, oxalic acid 200 g/dm^3^, acetanilide 200 g/dm^3^. 3: - glycerine 99% (50% *v*/*v*), H_3_PO_4_ 85% (35% *v*/*v*), H_2_O (15% *v*/*v*).	Surface roughness, chemical composition of the bath/316L, sample dimensions: 80 × 20 × 2 mm.	SS*: Ra* = 0.17–0.20 µm, Ra for bath 1 after EP*: Ra* = 0.063–0.065 µm, Ra for bath 2 after EP*: Ra* = 0.091–0.096 µm, Ra for bath 3 after EP (at 90 °C): *Ra* = 0.070 µm. Recommended parameters for baths 1 and 2: *t* = 15–20 min, *T* = 55 °C. Recommended parameters for bath 3: T = 90 °C.	[67]
*j* = 20 A/dm^2^, *T* = 55 °C, *t* = 12 min.	H_3_PO_4_ (51 wt.%), H_2_SO_4_ (35 wt.%), triethanolamine (3 wt.%), H_2_O (wt.% 11).	Surface roughness/bath contamination/304.	SS: *Ra =* 0.17–0.25 µm, pickled: *Ra =* 0.37–0.43 µm, after EP in industrial baths I and II: *Ra =* 0.14–0.20 µm (where iron content: 35–55 g Fe/dm^3^), after EP in industrial bath III: *Ra =* 0.17–0.28 µm (where iron content: 67–75 g Fe/dm^3^), after EP in laboratory bath with low iron content (3–6 g Fe/dm^3^): *Ra* < 0.11 µm.	[17]
*j* = 0.5–3.0 A/cm^2^, *T* = 50–95 °C, *t* = 3, 6, 9 min.	H_2_SO_4_: H_3_PO_4_ (vol.): 5:5, 4:6, 3:7, H_2_O, glycerine.	Surface roughness, corrosion resistance/316L Corrosion tests were conducted in FeCl_3_ solution (workpiece was put 2 cm deep). The container was sealed, and the temperature was controlled at 50 °C for 72 h.	*Rmax* = 0.8 µm, *Ra* = 0.08 µm (for recommended parameters), Recommended parameters: *j* = 1 A/cm^2^, *T* = 85 °C, *t* = 3–5 min, Bath composition: H_2_SO_4_; H_3_PO_4_—vol. 4:6, addition of 10% H_2_O.	[68]
*j* = 0.8 A/cm^2^, *T* = 40 °C, *t* = 420 s.	H_3_PO_4_ (64 wt.%), H_2_SO_4_ (13 wt.%), H_2_O (23 wt.%).	Surface roughness, corrosion resistance/316L For corrosion tests the saturated calomel electrode (SCE) was applied as the reference electrode and platinum foil as a counter electrode. Experiments were performed in physiological solution represented by 0.9 % NaCl solution.	SS*: Ra* = 0.256 µm, after EP: *Ra* = 0.078 µm, SS*: Rq* = 0.363 µm, after EP: *Rq* = 0.096 µm, SS*: Rz* = 2.290 µm, after EP*: Rz* = 0.474 µm.	[69]
*j* = 10, 29, 48, 67 A/dm^2^, *T* = 35, 45, 55, 65 °C, *t* = 3, 14, 25, 36 min.	1—H_2_SO_4_ 15%, H_3_PO_4_ 63%, H_2_O 22%,2—H_2_SO_4_ 35%, H_3_PO_4_ 45%, H_2_O 17%, CrO_3_ 3%,3—H_2_SO_4_ 35%, H_3_PO_4_ 45%, H_2_O 20%.	Surface roughness, chemical composition of the bath/316L.	Maximum *Ra* ranges after EP: 80–90% for: Recommended parameters: *j* = 48 A/dm^2^, *T* = 35 °C, *t* = 25 min, ∆*Ra* best in bath 3, worst in bath 1. Addition of CrO_3_ in bath 2 did not influence the electrochemical polishing result.	[70]
*j* = 30–50 A/dm^2^, *T* = 55 °C, *t* = 5–20 min, *S* = 4 cm^2^, *q* ≤ 167 mAh/cm^2^.	H_3_PO_4_ (51 wt.%), H_2_SO_4_ (35 wt.%), triethanolamine (3 wt.%), H_2_O (11 wt.%).	Corrosion resistance, chemical composition of the surface/304 During the corrosion tests, a tested electrode was 304 steel, reference saturated calomel electrode (SCE) was the electrode and the counter electrode was a platinum electrode.	SS: *Epit* = 0.30 V, pickled: *Epit* = 0.38 V, after EP *(*for *t* = 6 min, *j* = 30 A/dm^2^*): Epit* = 0.57 V, after EP (for *t* = 5 min, 20 min, *j* = 50 A/dm^2^): *Epit* (for 5 min) = 0.60 V, *Epit* (for 20 min) = 0.49 V, at.% ΣFe (FeO, Fe_2_O_3_, Fe_3_O_4_, FeOOH): SS*:* at.% ΣFe = 6.1%, pickled: at.% ΣFe = 11.9%, after EP: at.% ΣFe = 14.5%,at.% ΣCr (CrO_2_, Cr_2_O_3_, CrO_3_, Cr(OH)_3_): SS*:* at.% ΣCr = 18.0%, pickled: at.% ΣCr = 17.9%, after EP: at.% ΣCr = 28.6%.	[71,72]
*U* = 25 V, *T* = 30 °C, *t* = 20 s.	HClO_4_ 70% (20% *v*/*v*), CH_3_COOH 98% (80% *v*/*v*).	Corrosion resistance, chemical composition of the surface/316L, sample dimensions: 10 × 10 × 1 Corrosion tests were conducted at 30 °C in a solution with composition water, i.e., 1000 mg/L of B as H_3_BO_3_ and 2 mg/L of Li as LiOH. The reference electrode was a saturated calomel electrode (SCE), and a counter electrode was a platinum plate.	XPS analysis: after EP: at.% Cr (hydroxide) ≈ 35%, at.% Cr (oxide) ≈ 7%, mechanical processing: after CPS (polishing with colloidal silicate) at.% Cr (oxide) ≈ 30%, after EP: at.% Fe (hydroxide) ≈11%, at.% Fe (oxide) ≈ 25%, mechanical processing: after CPS: at.% Fe (oxide) ≈ 28%, corrosion: after EP: *Rs* = 8736 Ω·cm^2^, after CPS: *Rs* = 8840 Ω·cm^2^, after EP: *C1* = 78.2 µF/cm^2^, after CPS *C1* = 42.5 µF/cm^2^, after EP: *R1* = 0.41 Ω·cm^2^, after CPS *R1* = 0.97 Ω·cm^2^, after EP: *C2* = 63.8 µF/cm^2^, after CPS *C2* = 35.9 µF/cm^2^, after EP: *R2* = 3.04 Ω·cm^2^, after CPS *R2* = 5.69 Ω·cm^2^.	[73]
―	―	Corrosion resistance/316L, stents: Corrosion tests were conducted in tyrode solution at 37 °C constant temperature. Calomel electrode (SCE) as a reference electrode and platinum plate served as an auxiliary electrode.	Corrosion resistance: After mechanical processing: *Ecor* = −0.533 V, after EP: *Ecor* = −0.324 V, After mechanical processing: *Vcor* = 0.04 mm/year, after EP: *Vcor* = 0.119 mm/year.	[74,75]
*U* = 3 V, *j* = 1.25–25.5 A/dm^2^, *t* = 5–25 min.	H_3_PO_4_ (55 wt.%), H_2_SO_4_ (14 wt.%), H_2_O (31 wt.%).	Gloss, corrosion resistance, chemical composition, adhesion/304.	SS*:* Gloss G = 400, after EP: G = 1700–2500. Chemical composition: wt.% Cr increase after EP*:* from 18.83% to 19.33%.	[76]
*j* = 15.5; 31.0; 46.5 A/dm^2^, *T* = 333, 343, 353 K, *t* = 5–12 min.	1—H_3_PO_4_ (500 mL/L), H_2_SO_4_ (360 mL/L), monoethanolamine 20 (mL/L), 2—H_3_PO_4_ (500 mL/L), H_2_SO_4_ (360 mL/L), diethanolamine 20 mL/L, 3—H_3_PO_4_ (500 mL/L), H_2_SO_4_ (360 mL/L), triethanolamine 20 mL/L.	Gloss, bath composition/304, Sample dimensions: 25 × 25 × 1 mm.	Bath 1: (9 min, 31 A/dm^2^ at 333, 353K)-reflection coefficient = 98%, bath 2: (46.5 A/dm^2^)-max reflection coefficient 98%, bath 3: (9 and 12 min, 15.5 A/dm^2^, 333K or 343K and 353K)-reflection coefficient = 99%.	[5]
*j* = 50 ± 2, 1000 ± 10 A/dm^2^, *T* = 65 ± 5, 55 ± 5 °C.	1—H_3_PO_4_ (20% *v*/*v*), H_2_SO_4_ (80% *v*/*v*). 2—H_3_PO_4_ (80% *v*/*v*), H_2_SO_4_ (20% *v*/*v*).	Chemical composition of surface, Young modulus/304L, 316L, sample dimensions: 30 × 5 × 1 mm.	XPS analysis: Cr/Fe ratio after EP (1000 A/dm^2^) = 1.5 in bath 1, Cr/Fe ratio after EP (1000 A/dm^2^) = 2.7 in bath 2, Bath 1: Young modulus higher for EP (50A /dm^2^), than for EP (1000 A/dm^2^). Thickness of *h* layer for 316L steel, after EP (50 A/dm^2^): PO_4_^3−^ >> SO_4_^2−^ *h* = 10 nm, PO_4_^3−^ > SO_4_^2−^ *h* = 3 nm, PO_4_^3−^ *h* = 12 nm, after EP (1000 A/dm^2^): PO_4_^3−^ ≈ SO_4_^2−^ *h* = 7 nm, PO_4_^3−^ > SO_4_^2−^ *h* = 3 nm, PO_4_^3−^ *h* = 3 nm, Bath 2: Young modulus higher after EP (1000 A/dm^2^), than after EP (50 A/dm^2^). Thickness of *h* layer for 316L steel, after EP (50 A/dm^2^): PO_4_^3−^ >> SO_4_^2−^ *h* = 10 nm, PO_4_^3−^ > SO_4_^2−^ *h* = 5 nm, PO_4_^3−^ *h* = 10 nm, after EP (1000 A/dm^2^): PO_4_^3−^ = 3 nm, PO_4_^3−^ >> SO_4_^2−^ = 17 nm, PO_4_^3−^ = 15 nm.	[77,78]
*I* = 1 A, *j* = 12.9 A/dm^2^, *T* = 55 ± 5 °C, *S* = 7.78 cm^2^.	H_3_PO_4_, H_2_SO_4_, H_2_O.	Chemical composition of surface/316L, sample dimensions: 19 × 19 × 0.737 mm.	Increase in at.% Cr content in the top layer from 16% after mechanical processing to 20% after EP. Decrease in at.% Fe content in the top layer from 18% after mechanical processing to 10% after EP.	[79]
*j* = 0.29 A/dm^2^, *t* = 1 h.	H_3_PO_4_ (430 mL), H_2_SO_4_ (25 mL), CrO_3_ (30 g).	Chemical composition of the surface/316.	AES SS: at.% Fe = 27%, pickled: at.% Fe = 25%, sanded: at.% Fe = 20%, pickled + EP*:* at.% Fe = 16%, sanded + EP*:* at.% Fe = 30%, SS*:* at.% Cr = 7%, pickled: at.% Cr = 8%, sanded: at.% Cr = 5%, pickled + EP*:* at.% Cr = 3%, sanded + EP: at.% Cr = 16%, SS*:* at.% Ni = 4%, pickled: at.% Ni = 4%, sanded: at.% Ni = 3%, pickled + EP*:* at.% Ni ≤ 2%, sanded + EP*:* at.% Ni = 3%, sanded: at.% Si = 22%.SS*:* at.% C = 4%, pickled: at.% C = 15%, sanded: at.% C = 25%, SS*:* at.% O = 50%, pickled: at.% O = 45%, sanded: at.% O = 16%, pickled + EP: at.% O = 56%, sanded + EP: at.% O = 40%.	[80]
*U* = 40 V, *t* = 75 s.	HClO_4_ (20% *v*/*v*), CH_3_COOH (80% *v*/*v*).	Chemical composition of the surface/316L Sample dimensions: 12 × 5 × 3 mm.	EDS SS: at.% Fe = 33.21 ± 0.89%, after MP (mechanical processing)*:* at.% Fe = 34.72 ± 3.22%, after EP*:* at.% Fe = 31.95 ± 2.72%, SS*:* at.% Cr = 1.18 ± 0.03%, after MP*:* at.% Cr = 1.21 ± 0.02%, after EP*:* at.% Cr = 1.31 ± 0.19%, SS: at.% Ni = 2.32 ± 0.35%, after MP: at.% Ni = 2.72 ± 0.41%, after EP: at.% Ni = 4.63 ± 1.00% SS*:* at.% O = 63.12 ± 0.9%, after MP*:* at.% O = 61.35 ± 2.83%, after EP*:* at.% O = 62.10 ± 3.89%.	[81]
*j* = 500 A/dm^2^, 1000 A/dm^2^, *T* = 60 ± 1 °C.	H_3_PO_4_, H_2_SO_4_.	Chemical composition of the surface/316L Sample dimensions: 25 × 5 × 1 mm.	XPS: Cr/ΣE4 ratio (Fe, Cr, Ni, O) after EP (500 A/dm^2^): Cr/ΣE4 = 0.0807, Cr/ΣE4 ratio (Fe, Cr, Ni, O) after MEP (225/50 A/dm^2^): Cr/ΣE4 = 0.0397, Cr/ΣE4 ratio (Fe, Cr, Ni, O) after MEP (225/1000 A/dm^2^): Cr/ΣE4 = 0.0673.	[82,83]
*j* = 50 ± 2 A/dm^2^, 1000 ± 10 A/dm^2^, *T* = 65 ± 5, 75 ± 5 °C	1—H_3_PO_4_ (60% *v*/*v*), H_2_SO_4_ (40% *v*/*v*) 2—H_3_PO_4_ (40% *v*/*v*), H_2_SO_4_ (60% *v*/*v*)	Chemical composition of the surface/316L Sample dimensions: 30 × 5 × 1 mm.	XPS: Fe2p signal lower after EP (1000 A/dm^2^) in bath 1 than after EP (50 A/dm^2^), Min. Fe content (at.% Fe = 2.5%) after EP (1000 A/dm^2^) in bath 1, at.% Cr2p (Cr6+) after EP: (50 A/dm^2^) = 1.6%, at.% Cr2p (Cr6+) after EP: (1000 A/dm^2^) = 23.2%.	[84]
*U* = 10 V, *T* = 60 °C.	H_2_SO_4_, H_3_PO_4_—vol. 1:3.	Chemical composition of the surface/316LVM.	XPS: after EP*:* at.% Cr = 1.8%, after MEP (magnetic electrochemical polishing): at.% Cr = 10.1%, after EP: at.% Mo = 0.4%, after MEP: at.% Mo = 1.9%, after EP: at.% Fe = 15.4%, after MEP: at.% Fe = 9.7%, after EP: at.% S = 1.5%, after MEP: at.% S = 3.9%, after EP: at.% P = 17.2%, after MEP: at.% P = 16.4%.	[85,86]
*I* = 2.5 A, *j* = 65 ± 5 A/dm^2^, *T* = 65 ± 5 °C, *t* = 3 min, *S* = 3.854 cm^2^, *q* = 32 mAh/cm^2^.	H_3_PO_4_, H_2_SO_4_—vol. 4:6.	Chemical composition of the surface/430 SS, Sample dimensions: 30 × 5 × 1.22 mm.	XPS: after MP (mechanical processing) Cr/Fe ratio = 0.4, after EP*:* Cr/Fe ratio = 2.25, after EP (electrochemical polishing with mixing) Cr/Fe ratio = 0.96, after MEP (magnetic electrochemical polishing) Cr/Fe ratio = 0.7.	[87]
*j* = 50 ± 1 A/dm^2^, 1000 ± 10 A/dm^2^, *T* = 65 ± 5 °C, 55 ± 5 °C.	H_3_PO_4_ (20% *v*/*v*), H_2_SO_4_ (80% *v*/*v*).	Chemical composition of the surface/2205 duplex steel. Sample dimensions: 30 × 5 × 1 mm.	Fe2p signal lower after EP (1000 A/dm^2^) than after EP (1000 A/dm^2^), after EP (50 A/dm^2^) Cr/Fe ratio = 1.9, after EP (1000 A/dm^2^) Cr/Fe ratio = 1.7, after EP (50 A/dm^2^) P/S ratio = 0.5, after EP (1000 A/dm^2^) P/S ratio = 0.3.	[88]
*U* = 8 V, 9.5 V; t = 15 min, 20 min.	H_2_SO_4_ 96%, H_3_PO_4_ 85% —mas. 1:1.	Hardness/316L.	Vickers microhardness SS*: VHN* = 168.27 kg/cm^2^, after sanding *VHN* = 283 kg/cm^2^, after sanding and EP *VHN* < 205 kg/cm^2^.	[89]
*j* = 20 A/dm^2^, *T* = 55 ± 2 °C, *t* = 2–4 min *S* = 20 cm^2^, *q* = 7–13 mAh/cm^2^.	H_3_PO_4_ (35% *v*/*v*), H_2_SO_4_ (60.5% *v*/*v*), triethanolamine (4.5% *v*/*v*.).	Hardness/316L Sample dimensions: 80 × 20 × 2 mm.	Vickers microhardness *SS*: 188 HV, after MP: 318 HV, after EP samples were characterized by lower microhardness 230 HV.	[90]
*j* = 4–8 A/dm^2^, *T* = 35–55 °C, t = 15–45 min.	H_3_PO_4_, H_2_SO_4_, triethanolamine (3 wt.%).	Surface defects, baths containing/304 surface area of approx. in industrial conditions 33.3 dm^2^, in laboratory conditions 0.4 dm^2^.	Not optimal variants of process parameters (current density, temperature) led to the emergence of defects on the surface of the electrochemical polished samples. Poor quality of surface was reflected high roughness results, exceeding 0.24 µm and even reaching 0.55 µm, and low gloss values, below 500 GU. Recommended parameters: T = 35 °C, j = 8 A/dm^2^.	[46]

## References

[1] Clerc C., Datta M., Landolt D. (1984). On the theory of anodic levelling: Model experiments with triangular nickel profiles in chloride solution. Electrochim. Acta.

[2] Taylor E.J., Inman M. (2014). Electrochemical Surface Finishing. Electrochem. Soc. Interface.

[3] Bagdach S. (2002). Poradnik Galwanotechnika. Praca Zbiorowa.

[4] Kao P., Hocheng H. (2003). Optimization of electrochemical polishing of stainless steel by grey relational analysis. J. Mater. Process. Technol..

[5] Jeyashree G., Subramanian A., Vasudevan T., Mohan S., Venkatachalam R. (2000). Electropolishing of stainless steel. Bull. Electrochem..

[6] Dobrev T., Pham D.T., Dimov S. (2006). Electrochemical Polishing: A Technique for Surface Improvements after Laser Milling.

[7] Niveen J.A., Hussain M. Study of Electrochemical Polishing Applications in some alloys to obtain high surface finish. Proceedings of the 2012 International Conference on Industrial Engineering and Operations Management.

[8] Kaladhar M., Subbaiah K.V., Rao C.H.S. (2012). Machining of austenitic stainless steels—A review. Int. J. Mach. Mach. Mater..

[9] Basmaci G., Ay M. (2017). Optimization of Cutting Parameters, Condition and Geometry in Turning AISI 316L Stainless Steel Using the Grey-Based Taguchi Method. Acta Phys. Pol. A.

[10] Zhao O., Afshan S., Gardner L. (2017). Structural response and continuous strength method design of slender stainless steel cross-sections. Eng. Struct..

[11] Lee S.-J., Lai J.-J. (2003). The effects of electropolishing (EP) process parameters on corrosion resistance of 316L stainless steel. J. Mater. Process. Technol..

[12] Ziemniak S., Hanson M. (2002). Corrosion behavior of 304 stainless steel in high temperature, hydrogenated water. Corros. Sci..

[13] Ziemniak S.E., Hanson M., Sander P.C. (2008). Electropolishing effects on corrosion behavior of 304 stainless steel in high temperature, hydrogenated water. Corros. Sci..

[14] Yang G., Wang B., Tawfiq K., Wei H., Zhou S., Chen G. (2016). Electropolishing of surfaces: Theory and applications. Surf. Eng..

[15] Tam S., Loh N., Mah C., Loh N. (1992). Electrochemical polishing of biomedical titanium orifice rings. J. Mater. Process. Technol..

[16] Simka W., Nawrat G., Chłodek J., Maciej A., Winiarski A., Szade J., Radwański K., Gazdowicz J. (2011). Electropolishing and anodic passivation of Ti6Al7Nb alloy. Przemysł Chem..

[17] Lochyński P., Łyczkowska E., Pawełczyk A., Szczygieł B. (2012). Effect of bath exploitation on steel electropolishing process efficiency. Przemysł Chem.

[18] Nawrat G., Bołd T., Simka W., Waś J., Gonet M., Gardela A., Nieużyła Ł. (2012). The influence of surface treatment of coronary stents on their corrosion resistance. Ochr. Przed Korozją.

[19] Raman S.G.S., Padmanabhan K.A. (1995). Effect of electropolishing on the room temperature low-cycle fatigue bahaviour AISI 304LN stainless steel. Int. J. Fatigue.

[20] Nawrat G. (2010). Elektrochemiczne Metody Inżynierii Powierzchni, Monograph.

[21] (2009). PN-EN 1672:2009 Food Processing Machinery—Basic Concepts-Part 2: Hygiene Requirements.

[22] (2013). PN-EN 14630:2013 Non-Active Surgical Implants—General Requirements.

[23] Metz F.I. (1960). Electropolishing of metals. Ph.D. Thesis.

[24] Smith E.L., Abbott A.P., Ryder K.S. (2014). Deep Eutectic Solvents (DESs) and Their Applications. Chem. Rev..

[25] Jacquet P.A. (1935). Electrolytic Method for obtaining Bright Copper Surfaces. Nature.

[26] Lee S.J., Chen Y.H., Hung J.H. (2012). The Investigation of Surface Morphology Forming Mechanisms in Electropolishing Process. Int. J. Electrochem. Sci..

[27] Buhlert M., Eugen G. (2009). Elektropolieren.

[28] Datta M., Landolt D. (2000). Fundamental aspects and applications of electrochemical microfabrication. Electrochim. Acta.

[29] Landolt D., Chauvy P.-F., Zinger O. (2003). Electrochemical micromachining, polishing and surface structuring of metals: Fundamental aspects and new developments. Electrochim. Acta.

[30] Maltosz M. (1995). Modeling of impedance mechanisms in electropolishing. Electrochim. Acta.

[31] Hryniewicz T. (1989). Physico-Chemical and Technological Fundamentals of Electropolishing Steels (Fizykochemiczne i Technologiczne Podstawy Procesu Elektropolerowania Stali).

[32] Ciszewski A. (2008). Technologia Chemiczna, Procesy Elektrochemiczne.

[33] Lin C.-C., Hu C.-C. (2008). Electropolishing of 304 stainless steel: Surface roughness control using experimental design strategies and a summarized electropolishing model. Electrochim. Acta.

[34] Lin C.-C., Hu C.-C., Lee T.-C. (2009). Electropolishing of 304 stainless steel: Interactive effects of glycerol content, bath temperature, and current density on surface roughness and morphology. Surf. Coat. Technol..

[35] Hryniewicz T. (2007). Wstęp do Obróbki Powierzchniowej Biomateriałów Metalowych.

[36] https://www.hanser-elibrary.com/doi/pdf/10.12850/9783874803052.fm.

[37] Faust C.L., Makio S. (1949). Surface Preparation by Electropolishing. J. Electrochem. Soc..

[38] Lee E.S. (2000). Machining Characteristics of the Electropolishing of Stainless Steel (AISI 316L). Int. J. Adv. Manuf. Technol..

[39] Nazneen F., Galvin P., Arrigan D., Thompson M., Benvenuto P., Herzog G. (2011). Electropolishing of medical-grade stainless steel in preparation for surface nano-texturing. J. Solid State Electrochem..

[40] Jullien C., Benezech T., Carpentier B., Lebret V., Faille C. (2003). Identification of surface characteristics relevant to the hygienic status of stainless steel for the food industry. J. Food Eng..

[41] Zaborski S. (2007). Obróbka Elektrochemiczno-Ścierna: Podstawy i Zastosowania.

[42] Chen S., Tu G., Huang C.A. (2005). The electrochemical polishing behavior of porous austenitic stainless steel (AISI 316L) in phosphoric-sulfuric mixed acids. Surf. Coat. Technol..

[43] Bhuyan A., Gregory B., Lei H., Yee S.Y., Gianchandani Y.B. Pulse and DC Electropolishing of Stainless Steel for Stents and Other Devices. Proceedings of the IEEE Sensors 2005.

[44] Núñez P.J., Plaza E.G., Prada M.H., Coronel R.T., López P.J.N. (2014). Electrolyte Effect on the Surface Roughness Obtained by Electropolishing of AISI 316L Stainless Steel. Mater. Sci. Forum.

[45] Maitak G.P., Yudenkova I.N., Pasechnik M.G., Drozd N.A. (1976). Electrochemical Polishing Solution. USSR Patent.

[46] Lochyński P., Charazińska S., Łyczkowska-Widłak E., Sikora A. (2019). Electropolishing of Stainless Steel in Laboratory and Industrial Scale. Metals.

[47] Alekseev G.I., Zot’eva G.A., Golovanov V.N., Boitsova T.V., Kuznetsov E.A. (1973). Electrolyte for Polishing Stainless Steels. USSR Patent.

[48] Hensel K.B. (2000). Electropolishing. Met. Finish..

[49] Hryniewicz T. Surface Electrochemistry for Materials and Mechanical Engineering. Proceedings of the International Science Conference “Challenges to Civil and Mechanical Engineering in 2000 and Beyond”.

[50] Taguchi C. (2007). Electrolytic Solution for Use in Electropolishing Process for Stainless Steel. Japan Patent.

[51] Taguchi C. (2007). Electrolytic Solution to Be Used for Electrolytic Polishing Method for Stainless Steel. Japan Patent.

[52] Gellér Z.E., Albrecht K., Dobránszky J. (2008). Electropolishing of Coronary Stents. Mater. Sci. Forum.

[53] Faust C.L. (1982). Electropolishing-Stainless Steel. Met. Finish..

[54] Faust C.L. (1982). Electropolishing-Stainless Steel, Part I. Met. Finish..

[55] Faust C.L. (1982). Electropolishing-Stainless Steel, Part II. Met. Finish..

[56] Faust C.L. (1983). Electropolishing, Carbon and Low Alloy Steel, Part I. Met. Finish..

[57] Abbott A.P., Capper G., McKenzie K.J., Ryder K.S. (2006). Voltammetric and impedance studies of the electropolishing of type 316 stainless steel in a choline chloride based ionic liquid. Electrochim. Acta.

[58] Eliaz N., Nissan O. (2007). Innovative processes for electropolishing of medical devices made of stainless steels. J. Biomed. Mater. Res. Part A.

[59] Sojitra P., Engineer C., Kothwala D., Raval A., Kotadia H., Mehta G. (2010). Electropolishing of 316LVM Stainless Steel Cardiovascular Stents: An Investigation of Material Removal. Surface Roughness and Corrosion Behaviour. Trends Biomater..

[60] Haïdopoulos M., Turgeon S., Sarra-Bournet C., Laroche G., Mantovani D. (2006). Development of an optimized electrochemical process for subsequent coating of 316 stainless steel for stent applications. J. Mater. Sci. Mater. Electron..

[61] Zhao H., van Humbeeck J., Sohier J., de Scheerder I. (2003). Electrochemical Polishing of 316L Stainless Steel Slotted Tube Coronary Stents: An Investigation of Material Removal and Surface Roughness. Prog. Biomed. Res..

[62] Löber L., Flache C., Petters R., Kühn U., Eckert J. (2013). Comparison of different post processing technologies for SLM generated 316l steel parts. Rapid Prototyp. J..

[63] Lochyński P., Kowalski M., Szczygieł B., Kuczewski K. (2016). Improvement of the stainless steel electropolishing process by organic additives. Pol. J. Chem. Technol..

[64] Habibzadeh S., Li L., Shum-Tim D., Davis E.C., Omanovic S. (2014). Electrochemical polishing as a 316L stainless steel surface treatment method: Towards the improvement of biocompatibility. Corros. Sci..

[65] Rahman Z.U., Deen K., Cano L., Haider W. (2017). The effects of parametric changes in electropolishing process on surface properties of 316L stainless steel. Appl. Surf. Sci..

[66] Latifi A., Imani M., Khorasani M.T., Joupari M.D. (2013). Electrochemical and chemical methods for improving surface characteristics of 316L stainless steel for biomedical applications. Surf. Coat. Technol..

[67] Łyczkowska E., Lochyński P., Chlebus E. (2013). Electropolishing of a stainless steel. Przemysł Chem..

[68] Hocheng H., Kao P., Chen Y. (2001). Electropolishing of 316L Stainless Steel for Anticorrosion Passivation. J. Mater. Eng. Perform..

[69] Oravcová M., Palček P., Zatkalíková V., Tański T., Król M. (2017). Surface treatment and corrosion behaviour of austenitic stainless steel biomaterial. IOP Conf. Ser. Mater. Sci. Eng..

[70] Núñez P., García-Plaza E., Hernando M., Trujillo R. (2013). Characterization of Surface Finish of Electropolished Stainless Steel AISI 316L with Varying Electrolyte Concentrations. Procedia Eng..

[71] Lochyński P., Łyczkowska E., Kuczewski K., Szczygieł B. (2014). Pitting corrosion of pickled and electropolished Cr-Ni stainless steel. Przemysł Chem..

[72] Lochyński P., Sikora A., Szczygieł B. (2016). Surface morphology and passive film composition after pickling and electropolishing. Surf. Eng..

[73] Han Y., Mei J., Peng Q., Han E.H., Ke W. (2016). Effect of electropolishing on corrosion of nuclear grade 316L stainless steel in deacerated high temperature water. Corros. Sci..

[74] Baron A., Simka W., Nawrat G., Szewieczek D., Krzyżak A. (2006). Influence of electrolytic polishing on electrochemical behaviour of austenitic steel. J. Achiev. Mater. Manuf. Eng..

[75] Baron A., Simka W., Nawrat G., Szewieczek D. (2008). Electropolishing and chemical passivation of austenitic steel. J. Achiev. Mater..

[76] Awad A., Ghazy E., El-Enin S.A., Mahmoud M. (2012). Electropolishing of AISI-304 stainless steel for protection against SRB biofilm. Surf. Coat. Technol..

[77] Rokosz K., Hryniewicz T., Lukeš J., Sepitka J. (2015). Nanoindentation studies and modeling of surface layers on austenitic stainless steels by extreme electrochemical treatments. Surf. Interface Anal..

[78] Rokosz K., Lahtinen J., Hryniewicz T., Rzadkiewicz S. (2015). XPS depth profiling analysis of passive surface layers formed on austenitic AISI 304L and AISI 316L SS afterhigh-current-density electropolishing. Surf. Coat. Technol..

[79] Selvaduray G., Trigwell S. Effect of Surface Treatment on the Surface Characteristics of AISI 316L Stainless Steel. Proceedings of the Conference of Materials and Processes for Medical Devices.

[80] Rao T.V. (1986). Effect of surface treatments on near surface composition of 316 nuclear grade stainless steel. J. Vac. Sci. Technol. A.

[81] Han G., Lu Z., Ru X., Chen J., Xiao Q., Tian Y. (2015). Improving the oxidation resistance of 316L stainless steel in simulated pressurized water reactor primary water by electropolishing treatment. J. Nucl. Mater..

[82] Hryniewicz T., Rokosz K. (2010). Analysis of XPS results of AISI 316L SS electropolished and magnetoelectropolished at varying conditions. Surf. Coat. Technol..

[83] Hryniewicz T., Rokosz K., Micheli V. (2011). Auger/AES surface film measurements on AISI 316L biomaterial after magnetoelectropolishing. Pomiary Autom. Kontrola.

[84] Rokosz K., Hryniewicz T., Rzadkiewicz S., Raaen S. (2015). High-current-density electropolishing (HDEP) of AISI 316L (EN 1.4404) stainless steel. Tehnički vjesnik.

[85] Rokosz K., Hryniewicz T., Rokicki R. (2014). XPS Measurements of AISI 316LVM SS Biomaterial Tubes after Magnetoelectropolishing. Tehnički vjesnik.

[86] Rokosz K., Hryniewicz T., Raaen S. (2014). Cr/Fe Ratio by XPS Spectra of Magnetoelectropolished AISI 316L SS Fitted by Gaussian-Lorentzian Shape Lines. Tehnički vjesnik.

[87] Hryniewicz T., Rokosz K., Raaen S. (2012). XPS Measurements of AISI 430 SS Surface after Electropolishing Operations, in a Transpassive Region of Polarisation Characteristics. Pomiary Autom. Kontrola.

[88] Rokosz K., Hryniewicz T., Simon F., Rzadkiewicz S. (2016). Comparative XPS analyses of passive layers composition formed on Duplex 2205 SS after standard and high-current-density electropolishing. Tehnički vjesnik.

[89] Suyitno S. (2014). The Influence of Sandblasting and Electropolishing on the Surface Hardness of AISI 316L Stainless Steel. Adv. Mater. Res..

[90] Lyczkowska-Widlak E., Lochyński P., Nawrat G., Chlebus E. (2019). Comparison of electropolished 316L steel samples manufactured by SLM and traditional technology. Rapid Prototyp. J..

[91] Arnold J.W., Boothe D.H., Suzuki O., Bailey G.W. (2004). Multiple imaging techniques demonstrate the manipulation of surfaces to reduce bacterial contamination and corrosion. J. Microsc..

[92] Shih C.-C., Shih C.-M., Su Y.-Y., Su L.H.J., Chang M.-S., Lin S.-J. (2004). Effect of surface oxide properties on corrosion resistance of 316L stainless steel for biomedical applications. Corros. Sci..

[93] Harris L., Meredith D.O., Eschbach L., Richards R.G. (2007). Staphylococcus aureus adhesion to standard micro-rough and electropolished implant materials. J. Mater. Sci. Mater. Electron..

[94] Verran J., Rowe D., Cole D., Boyd R. (2000). The use of the atomic force microscope to visualise and measure wear of food contact surfaces. Int. Biodeterior. Biodegrad..

[95] Gündüz G.T., Tuncel G. (2006). Biofilm formation in an ice cream plant. Antonie Leeuwenhoek.

[96] Poulsen L.V. (1999). Microbial Biofilm in Food Processing. LWT-Food Sci. Technol..

[97] Bryers J.D. (1994). Biofilms and the technological implications of microbial cell adhesion. Colloids Surf. B Biointerfaces.

[98] Zottola E.A. (1994). Microbial attachment and biofilm formation: A new problem for the food industry. Food Technol..

[99] Myszka K., Czaczyk K. (2011). Bacterial Biofilms on Food Contact Surfaces—A Review. Pol. J. Food Nutr. Sci..

[100] Lechevallier M.W., Babcock T.M., Lee R.G. (1987). Examination and characterization of distribution system biofilms. Appl. Environ. Microbiol..

[101] Lochynski P., Charazińska S., Łyczkowska-Widłak E., Sikora A., Karczewski M. (2018). Electrochemical Reduction of Industrial Baths Used for Electropolishing of Stainless Steel. Adv. Mater. Sci. Eng..

[102] Gadali’ nska E., Wronicz W. (2016). Electropolishing procedure dedicated to in-depth stress measurements withx-ray diractometry. Fatigue Aircr. Struct..

